# GeoCENS: A Geospatial Cyberinfrastructure for the World-Wide Sensor Web

**DOI:** 10.3390/s131013402

**Published:** 2013-10-02

**Authors:** Steve H.L. Liang, Chih-Yuan Huang

**Affiliations:** Department of Geomatics Engineering, University of Calgary, 2500 University Drive NW, Calgary, AB T2N 1N4, Canada; E-Mail: huangcy@ucalgary.ca

**Keywords:** GIS, sensor web, cyberinfrastructure, data interoperability, open geospatial consortium, sensor observation service

## Abstract

The world-wide sensor web has become a very useful technique for monitoring the physical world at spatial and temporal scales that were previously impossible. Yet we believe that the full potential of sensor web has thus far not been revealed. In order to harvest the world-wide sensor web's full potential, a geospatial cyberinfrastructure is needed to store, process, and deliver large amount of sensor data collected worldwide. In this paper, we first define the issue of the *sensor web long tail* followed by our view of the world-wide sensor web architecture. Then, we introduce the Geospatial Cyberinfrastructure for Environmental Sensing (GeoCENS) architecture and explain each of its components. Finally, with demonstration of three real-world *powered-by-GeoCENS* sensor web applications, we believe that the GeoCENS architecture can successfully address the sensor web long tail issue and consequently realize the world-wide sensor web vision.

## 1. Introduction

In recent years, large-scale sensor arrays and the vast datasets that are produced worldwide are being utilized, shared and published by a rising number of researchers on an ever-increasing frequency. Examples include the global scale ARGOS network of buoys (http://www.argos-system.org), the weather networks of the World Meteorological Organization, and the global GPS Zenith Total Delay (ZTD) observation network. A significant amount of effort (e.g., GEOSS (http://www.earthobservations.org/geoss.shtml) and NOAA IOOS (http://ioos.gov/)) has been put forth to web-enable these large-scale sensor networks so that the sensors and their data can be accessible through interoperable sensor web standards. Moreover, with the advent of the low-cost sensor networks and data loggers, it is technologically and economically feasible for individual scientists to deploy and operate small- to medium-scale sensor arrays. Individual scientists or small research groups can now easily deploy multiple sensor arrays at strategic locations for their own research purposes. There is a spectrum of sensor networks ranging from local-scale short-term sensor arrays to global-scale permanent observatories. The vision of the *World-Wide Sensor Web* is becoming a reality.

The original world-wide sensor web concept was proposed by the NASA/Jet Propulsion Laboratory (JPL) in 1997 [1] for acquiring environmental information by integrating massive spatially distributed consumer-market sensors. With the development of sensor technology, the sensor web concept has become broader than NASA's original definition and is more related to the concepts of web-enabling sensor networks [2]. The sensor web/network is increasingly attracting the interest of researchers for a wide range of applications. These include: large-scale environmental monitoring [3–5], civil structures [6], roadways [7,8], and animal habitats [9,10]. Sensor web applications range from video camera networks that monitor real-time traffic to matchbox-sized wireless sensor networks embedded in the environment to monitor habitats. The world-wide sensor web generates tremendous volumes of priceless streaming data that enables scientists to observe previously unobservable phenomena.

Similar to the World-Wide Web (WWW), which acts essentially as a “World-Wide Computer”, the sensor web can be considered as a “World-Wide Sensor” or a “cyberinfrastructure”. This World-Wide Sensor is capable of monitoring the physical world at spatial and temporal scales that were previously impossible. However, harvesting the full potential of sensor web is very challenging. In order to build a sensor web system, we need to address the issue of the *world-wide sensor web long tail*, where sensor data produced by smaller organizations or individuals are unavailable to the public.

The preliminary idea of GeoCENS was first presented as a long abstract (2,292 words) in the 6th International Conference on Geographic Information Science [11]. The long abstract is not included in the paper proceedings and only describes GeoCENS superficially. On the other hand, this journal paper was written to fully communicate the GeoCENS architecture, in which we provide complete background introduction, high-level solutions (with references to detailed algorithms), real-world applications, and comparison with related systems.

The remainder of this paper is organized as follows. Section 2 explains the long tail phenomenon in the sensor web. In Section 3, we present our view of the world-wide sensor web architecture. Section 4 presents the proposed GeoCENS architecture as one possible solution, including its key components and algorithms. While Section 5 introduces the real-world sensor web applications that utilize the GeoCENS architecture, Section 6 lists the previous works that are related to this research. Finally, Section 7 will address conclusions and future work.

## 2. The World-Wide Sensor Web Long Tail

**Figure 1.**
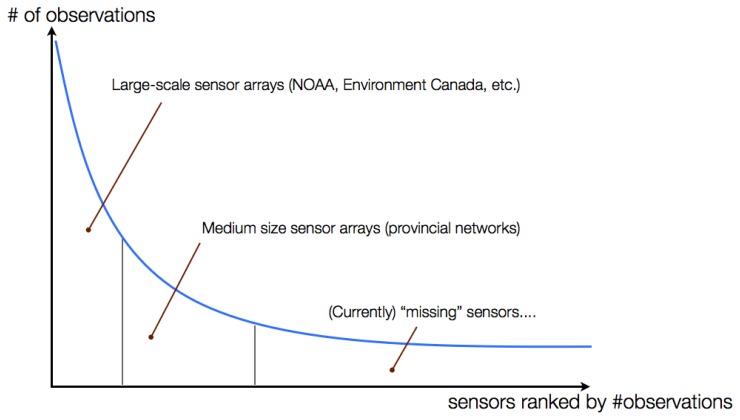
The sensor web long tail.

The concept of sensor web is to connect all the sensors in the world and their data together to achieve shared goals [12]. A major objective of sensor web is to improve the openness and accessibility of sensor data, which we refer to this as the *open data for sensor web*. However, the *long tail phenomenon in the sensor web* that we have previously observed [13] could cause issues on the open data for sensor web vision. As shown in Figure 1, we divide the long tail into three parts, namely the *head*, the *middle*, and the *tail*. The head mainly consists of large scale sensor arrays operated by national organizations, such as NOAA and Environment Canada. Although the number of these large organizations is small, they collect a vast amount of sensor data. The middle contains medium size sensor arrays, which are usually maintained by provincial organizations. Compared to the head, the middle has more operating organizations but a smaller amount of sensor data. The sensor data in the tail are collected by small organizations or individuals, such as small research groups and scientists. However, unlike the head and the middle, the small organizations and individuals in the tail usually do not maintain sensor arrays for a long period of time; instead, they collect data based on the need of their short term projects. Therefore, although there are a large number of participants in the tail, the amount of sensor data collected by each of them is small.

Based on the definition of the long tail [14], we know that the summation of the sensor data in the tail has a similar size to that in other parts. However, most of the sensor data in the tail are usually not accessible to the public due to the lack of interoperable and easy-to-use ways to share the data online. Hence, we call the sensors in the tail as *missing sensors* or *dark sensors*. In order to address the interoperability issue that constitute an obstacle to sharing sensor data online, the Open Geospatial Consortium (OGC) Sensor Web Enablement (SWE) working group defines open standard protocols and data models for sensor devices and sensor data [15,16]. The OGC SWE standards are similar to the open standards defined by the World Wide Web Consortium (W3C) for the World Wide Web (WWW) that provide an interoperable way for people to communicate on the Internet. Therefore, in order to *capture the long tail* and achieve the vision of open data for sensor web, a cyberinfrastructure that allows organizations or individuals to easily share their sensor data through the OGC SWE standards is one of the necessary components.

However, similar to that of the WWW, in addition to open standards, many other necessary components need to be considered in order to harvest the full potential of sensor web. In the following section, we introduce our view of the sensor web architecture to further analyze these necessary components.

## 3. The World-Wide Sensor Web Architecture

To the best of our knowledge about current sensor web development, we envision the architecture of the world-wide sensor web would be very similar to that of the WWW. For example, the WWW connects all of the web services around the world through open standard protocols (e.g., HTTP), which has been proven to be very scalable in terms of interchanging messages worldwide. The current sensor web development is moving toward a similar direction. We can see this trend from many sensor web projects, such as SensorWare Systems (http://www.sensorwaresystems.com/), Microsoft SensorWeb project (http://research.microsoft.com/en-us/projects/senseweb/), and Xively.com (https://xively.com/). These projects deploy sensors, collect sensor data, and host and share the data on the WWW through proprietary protocols.

**Figure 2.**
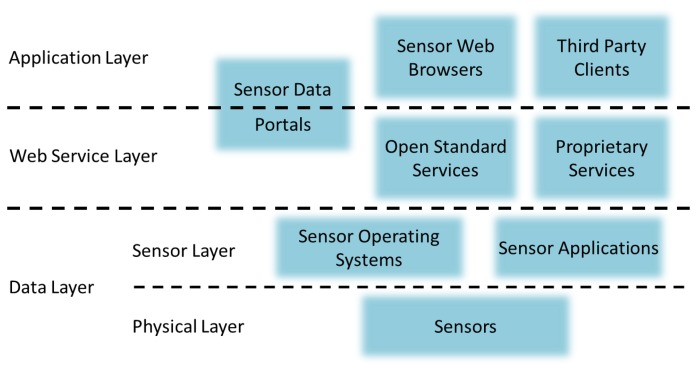
The sensor web layer stack.

Similar to the WWW, the sensor web mainly has three layers, namely, the data layer, the web service layer, and the application layer. The sensor web layer stack is shown in the Figure 2. The data layer can be further divided into the physical layer and the sensor layer. While the data layer performs observations (here we follow OGC SWE's definition of observation, which is “*an act of observing a property or phenomenon, with the goal of producing an estimate of the value of the property*”) and transmits sensor data to the web service layer, the web service layer provides the access for the application layer to retrieve the cached sensor data.

Since the architectures of sensor web and WWW would be very similar, the components that are essential for the WWW should be considered in the development of sensor web. For example, here we identify three high-level components that are essential for the current WWW, namely, the open standard protocols, the resource discovery services, and the client-side platforms.

1. *Open Standard Protocols*: Open standard protocols play one of the most important roles in the success of WWW. The communications in the Internet layers, such as the seven layers in the OSI model (ISO/IEC 7498-1) and the four layers in the TCP/IP model (IETF RFC 1122), are handled with open standard protocols. For example, the IEEE 802 standards (http://standards.ieee.org/about/get/802/802.html) define protocols for the local area networks (LAN), including the Ethernet and the wireless LAN. The Internet Protocol (IP) defines the format of Internet packets and provides an addressing system for routing packets from a source host to a destination host. The Transmission Control Protocol (TCP) and User Datagram Protocol (UDP) are the two commonly used standards in the transport layer. Furthermore, the Hypertext Transfer Protocol (HTTP) is a protocol in the application layer that controls the high-level communications between applications. For example, a user may use a web browser, a client-side application, to send an HTTP request to an application running on a server hosting a web site. Then, the server could return resources, such as Hypertext Markup Language (HTML) files, in a HTTP response to the client.
  These open standards (and many others) developed by the Internet Engineering Task Force (IETF), the WWW Consortium (W3C), and the ISO/International Electrotechnical Commission (IEC) make sure the Internet components are interoperable. These groups have had a significant contribution in the success of the WWW. To prevent “reinventing the wheel”, the current sensor web development is built on top of many existing WWW standards. However, as content on the sensor web is fundamentally different from that on the WWW, additional open standard protocols should be defined. The OGC has defined many open standards for the sensor web community, including standards for data models, encodings, and web service interfaces. Although these standards are not as popular as the WWW standards, the development and adaption of open standards for the sensor web is one of the necessary steps to realize the sensor web vision.
2. *Resource Discovery Services*: As the open standard protocols handle the communications between web services located worldwide, resource discovery is a critical issue considering the highly distributed nature of the WWW. In the current WWW, the search engines (e.g., Google, Bing, Yahoo!) and the web portals (e.g., Yahoo!, MSN, CNN.com) were developed to address the resource discovery issue and to help users find the resources of interest. In addition, because sensor web services can be as distributed as WWW services, the sensor web resource discovery issue must be addressed as well. OGC defines the Catalogue Service for the Web standard (CSW) for users to discover sensor data and sensor web services by querying their metadata [17]. The Sensor Instance Registry (SIR) is another solution for addressing the sensor web resource discovery issue [18]. As SIR is currently under discussion in the OGC sensor web community, it defines web service interfaces for users to insert, describe, and search for sensors in SWE services. However, for both CSW and SIR solutions, service providers or users need to register SWE services to CSW or SIR services in the first place. Therefore, we believe that the resource discovery for sensor web is one of the remaining issues to be solved.
3. *Client-side Platforms:* Nowadays, client-side platforms allowing users to send requests and visualize responses have become an essential part of the WWW. The most popular type of client-side platforms are web browsers (e.g., Internet Explorer, Chrome, Firefox). As long as web browsers and web services follow the same protocols (e.g., HTTP and HTML), users can use web browsers to communicate with web services and visualize the response content (e.g., texts, images, videos). For the sensor web, we have observed that the most common client-side platforms are *sensor data portals*, which serve as intermediaries between users and the sensor data they host. Sensor data portals have full knowledge about the data they host (e.g., sensor locations and sampling times). Knowledge about hosted sensor data can then be used to pre-generate indices with the spatio-temporal distribution of sensor data to optimize the data transmission. For example, Ahmad and Nath [19] proposed the COLR-Tree to aggregate and sample sensor data to reduce data size before transmission. Some sensor data portals, e.g., the Groundwater Information Network (GIN) (http://analysis.gw-info.net/gin/public.aspx), present a map of sensor locations at small scale and actual sensor observations at large scale, which limits the number of sensor observations being transmitted in each request. However, a critical drawback of these sensor data portals is that they can only present the sensor data about which they have prior knowledge. Instead, we envision that a client-side platform for the sensor web should be capable of communicating with any sensor web services as long as clients and services follow the same protocol. As this type of client-side platform does not require any prior knowledge of the data to retrieve and visualize sensor data, we term this pure client-side application as *sensor web browsers*.
4. *Additional Components*: Besides the previous three essential components, some other ideas on the WWW may be helpful for the sensor web as well. For example, the online social network may be helpful for recommending sensor data to users according to their interests. In addition, as Web 2.0 is one of the fundamental concepts of volunteered geographic information (VGI) [20] and has demonstrated its usefulness (e.g., the Open Street Map), the sensor web can certainly benefit from the Web 2.0 concept by also capturing the data produced by volunteer citizens (*i.e.*, “human sensors”). Moreover, one of the important and ongoing directions of the WWW is the semantic web (http://www.w3.org/standards/semanticweb/), which aims to convert the unstructured and semi-structured content on the current WWW into a *web of data* using technologies that include W3C-recommended languages for formalizing semantics of data (e.g., RDF, OWL), ontologies for organizing classes and properties, semantic annotation frameworks to identify and map instance data into ontology classes [21], as well as reasoning engines to infer complex facts from basic data items (e.g., Jena and Jess for rule-based reasoning). Although the semantic web still seems premature, where for example techniques for reasoning with streaming data are still needed, we believe the sensor web can also apply the semantic web techniques and technologies to integrate the heterogeneous sensor data.

As now, we have identified the necessary components for the sensor web. However, there are still many challenges to be solved, especially considering the geospatial nature of sensor data. For example, transmitting large volumes of sensor data across networks, efficiently retrieving and updating high-velocity sensor data streams, and effectively integrating heterogeneous sensor data are some of the important challenges. In order to address these challenges and realize the sensor web vision, we propose the Geospatial Cyberinfrastructure for Environmental Sensing (GeoCENS) architecture.

## 4. The GeoCENS Architecture

**Figure 3.**
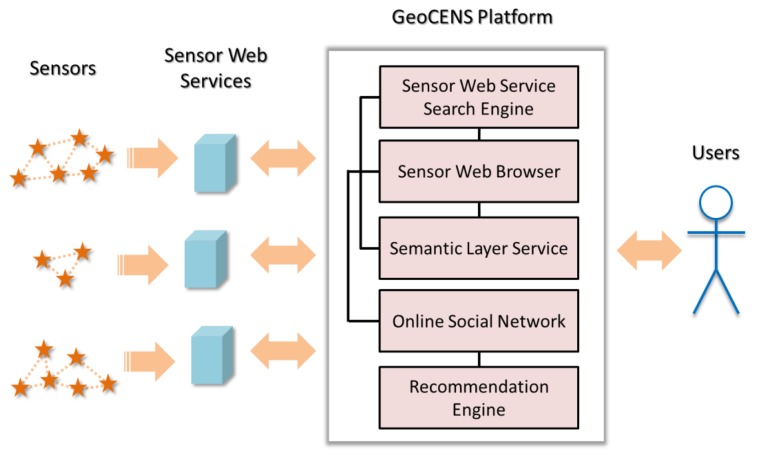
The GeoCENS architecture.

In the GeoCENS project, we design an architecture and build an online platform for the sensor web. GeoCENS allows users to maneuver a sensor web browser, within a 3D virtual globe or on a 2D base map, to discover, visualize, access and share heterogeneous and ubiquitous sensing resources, and as well as relevant information. Our aim is to address the aforementioned technical challenges, propose innovative approaches, and provide the missing software components for realizing the world-wide sensor web vision. Figure 3 shows the GeoCENS architecture.

Similar to that in the WWW, everyone can build and deploy sensor web services to host sensor data. Sensor web services may be distributed worldwide and are not registered on any catalogue service. GeoCENS proposes the *sensor web service search engine* to discover and index sensor web services, and allow users to search these services with query criteria. GeoCENS also develops a pure client-side *sensor web browser* application for users to retrieve and visualize sensor data from sensor web services. In addition, the *semantic layer service* utilizes the metadata in the sensor web services to integrate the heterogeneous sensor data layers to provide users a coherent view on the sensor web data. Furthermore, GeoCENS has an *online social network* component that allows users to establish friendships and share sensor data. With the friendship links on the online social network, GeoCENS provides the *recommendation engine* which has the ability to recommend sensors and datasets according to a user's interests. In the next sub-sections, we provide details of the various components of GeoCENS.

### 4.1. OGC-Based Sensor Web Servers

GeoCENS uses the OGC SWE open standards as the fundamental interoperability architecture. The availability of sensor web service implementations is very important for sensor data owners to easily install and share their sensor data in an interoperable manner. For example, the 52°North SOS (http://52north.org/communities/sensorweb/sos/), the MapServer SOS (http://mapserver.org/ogc/sos_server.html), and the Deegree SOS (http://wiki.deegree.org/deegreeWiki/deegree3/Sensor ObservationService) implementations are available for public to download and install. GeoCENS has also implemented the OGC Sensor Observation Service specification (SOS) version 1.0 [22], SensorML specification [23], and Observation and Measurement specification (O&M) [24]. The GeoCENS SOS implementation has been released online (http://wiki.geocens.ca/sos) to help sensor data owners easily deploy their sensor web services.

### 4.2. Decentralized Hybrid P2P Sensor Web Service Discovery

For any large-scale distributed system (e.g., the WWW), both communication and data management distill down to the problem of resource discovery. Similarly, GeoCENS needs a sensor web resource discovery service. In order to handle sensor web's large numbers of sensors and large numbers of users, GeoCENS uses a hybrid P2P architecture for sensor web resource discovery. Every GeoCENS sensor web server also serves as part of the sensor web service discovery infrastructure (*i.e.*, a peer node). These nodes operate on a cooperative model, where each peer leverages each other's available resources (*i.e.*, CPU, storage, bandwidth, *etc.*) for mutual benefit.

From the literature and existing systems, there are two types of P2P architectures: unstructured P2P networks, e.g., CAN [25], Pastry [26], and Chord [27] and structured P2P networks, e.g., Gnutella [28]. Nodes participating in unstructured P2P networks perform actions for each other, where no rules exist to define or constrain connectivity between nodes. The unstructured P2P networks are simple but not scalable because their flood-based query processing generates enormous amounts of network traffic. Structured P2P networks use hash functions to build distributed indexes for their stored data items. The hash tables, like distributed indexes, successfully reduce the number of nodes scanned per query. However, structured P2P networks are vulnerable to node dynamics.

GeoCENS proposes a hybrid approach that uses both structured and unstructured P2P networks [29]. The rationale of such hybrid design is described as follows. We envision that the future sensor web will have two types of sensor web servers. (1) Powerful sensor web servers maintained by large institutions (e.g., NASA or NOAA). These servers would not join and leave the network randomly and in most cases are always made accessible. Near constant accessibility means these servers act as static nodes in the network. (2) Less powerful sensor web servers maintained by small institutions or even individuals (e.g., universities or citizen scientists). These servers might join and leave the network more frequently, acting as dynamic and transient nodes in the network. Considering the above-described settings, it is a rational design decision to group static P2P nodes into structured super-nodes (to exploit the stability of static nodes) and group dynamic P2P nodes into leaf-nodes (to save the overhead for maintaining the structure).

Since structured P2P networks can only process exact key-value pair queries, we enable geospatial search functions by labeling data with space filling curves. For example, a geographical location (*i.e.*, latitude and longitude) can be converted from a 2D coordinate into a one-dimensional string (*i.e.*, quadkey) using Peano space filling curves with a particular level of detail. And the one-dimensional strings can be used to perform geospatial searches. This architecture is also unique in that it is a locality-aware system. The system is able to exploit the locality information between peer nodes in order to deliver the query results quickly and efficiently.

**Figure 4.**
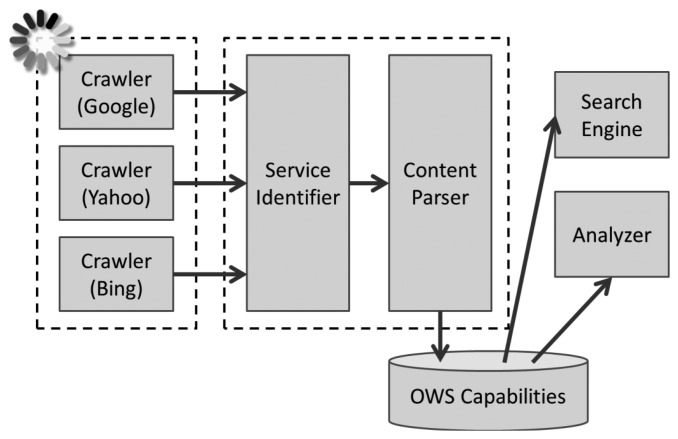
The GeoCENS search engine architecture.

The GeoCENS search engine is also able to discover non-GeoCENS OGC web services (OWS), which are not on the P2P overlay network. The GeoCENS search engine implements crawlers to periodically look for and index online OWS services. Therefore, users can still be able to find these services through the GeoCENS search engine. The architecture of the GeoCENS search engine is shown in Figure 4. Currently, the GeoCENS search engine has discovered 2,884 WMS services, which have 88,281 WMS layers, and 36 SOS services, which have 5,310 SOS observation offerings and 39,368 sensors/procedures. Please note that currently GeoCENS only supports SOS version 1.0. SOS version 2.0 support is a future work item.

### 4.3. 3D Virtual-Globe-Based and 2D Map-Based Sensor Web Browsers

The GeoCENS sensor web browser is an intuitive 3D client frontend for all OGC SOS services and OGC Web Map Service (WMS) [30]. It allows users to maneuver a 3D sensor web browser, within a single virtual globe. A user can browse, discover, visualize, access, share and tag heterogeneous sensing resources and other relevant information. Starting from a “zoomed out” view of the globe, users are able to select a study site and “fly” into it. While flying to their study sites, multiple resolutions of map data can be loaded to the client from the WMS servers. The GeoCENS browser combines multiple sensor data streams and geographical datasets, and render them in a coherent and unified virtual globe environment.

**Figure 5.**
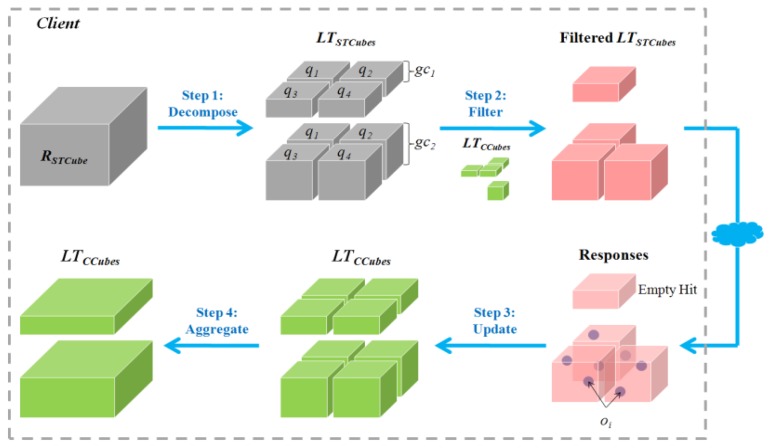
The LOST-Tree workflow.

**Figure 6.**
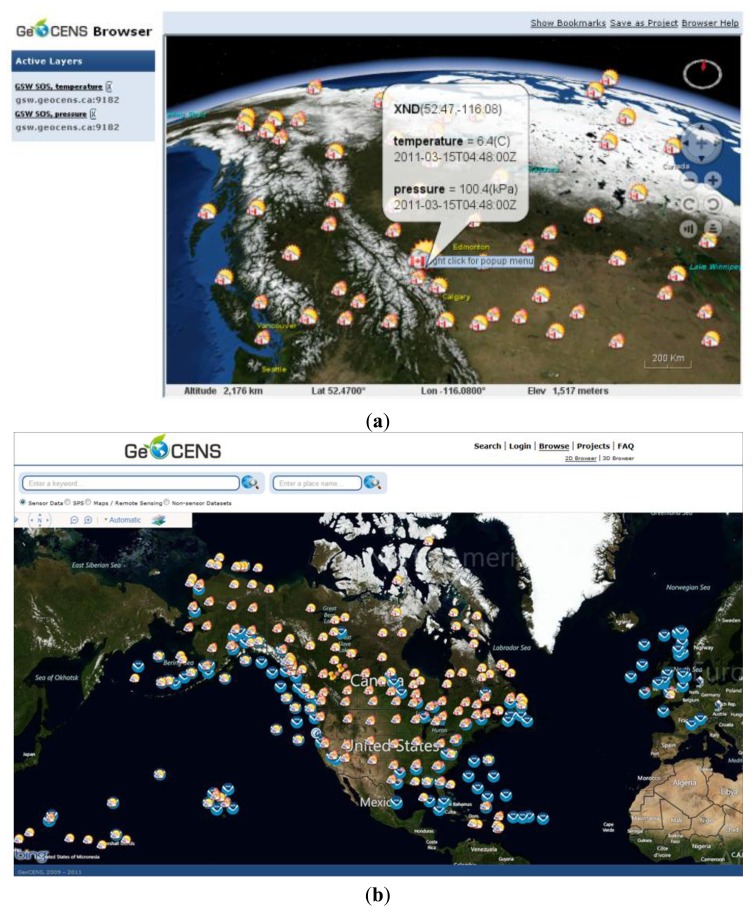
Screenshots of (**a**) 3D virtual-globe-based and (**b**) 2D map-based sensor web browsers.

The GeoCENS sensor web browser was developed on top of the open source WorldWind virtual globe system (http://worldwind.arc.nasa.gov/). To the best of our knowledge, it is the world's first OGC-based sensor web 3D browser. The GeoCENS browser has two unique components/contributions. (1) In order to interoperate with existing sensor web servers, an OGC SWE communication module was developed to communicate with OGC SWE-compatible servers. (2) In order to prevent transferring large volume of sensor data across the network repeatedly, a new spatio-temporal data loading module was developed. This new module was named LOST-Tree [31] and it utilized a client-side cache. LOST-Tree applies predefined hierarchical spatial and temporal frameworks to index requests instead of responses. This allows LOST-Tree to become scalable regarding the number of sensor observations. As shown in Figure 5, LOST-Tree mainly has four steps. First, LOST-Tree decomposes user's spatio-temporal query (*R_STCube_*) into indexed spatio-temporal cubes (*LT_STCubes_*). Then LOST-Tree filters out the spatio-temporal cubes that have been loaded and cached locally (*LT_CCubes_*) from the *LT_STCubes_*. As the sensor data in *LT_CCubes_* can be loaded from the local cache, the data in the filtered *LT_STCubes_* will be retrieved from the service. After retrieving the data from the services, LOST-Tree updates the *LT_CCubes_* and aggregates these loaded spatio-temporal cubes/indices to reduce the memory footprint. A screenshot of the 3D virtual-globe-based sensor web browser is shown in Figure 6a.

In addition to the 3D virtual-globe-based sensor web browser, GeoCENS also develops a light-weight 2D map-based sensor web browser. The 2D sensor web browser retrieves sensor data cache from a mediator named as the *translation engine* [32]. As the translation engine handles the heavy communication load (e.g., SOAP and XML) with the sensor web services, the 2D map-based sensor web browser can retrieve the cached sensor data from the translation engine in a light-weight and efficient manner. This efficient data retrieval also makes this 2D map-based sensor web browser mobile-friendly. A screenshot of the 2D map-based sensor web browser is shown in the Figure 6b.

In order to update the cached data in a timely manner, the translation engine utilizes the *adaptive feeder* [33]. The adaptive feeder detects the data updating frequency on the sensor web services and fetches the latest sensor data from the services by adaptively scheduling requests. In this case, the cached sensor data in the translation engine can be always updated.

### 4.4. Online Social Network (OSN)

**Figure 7.**
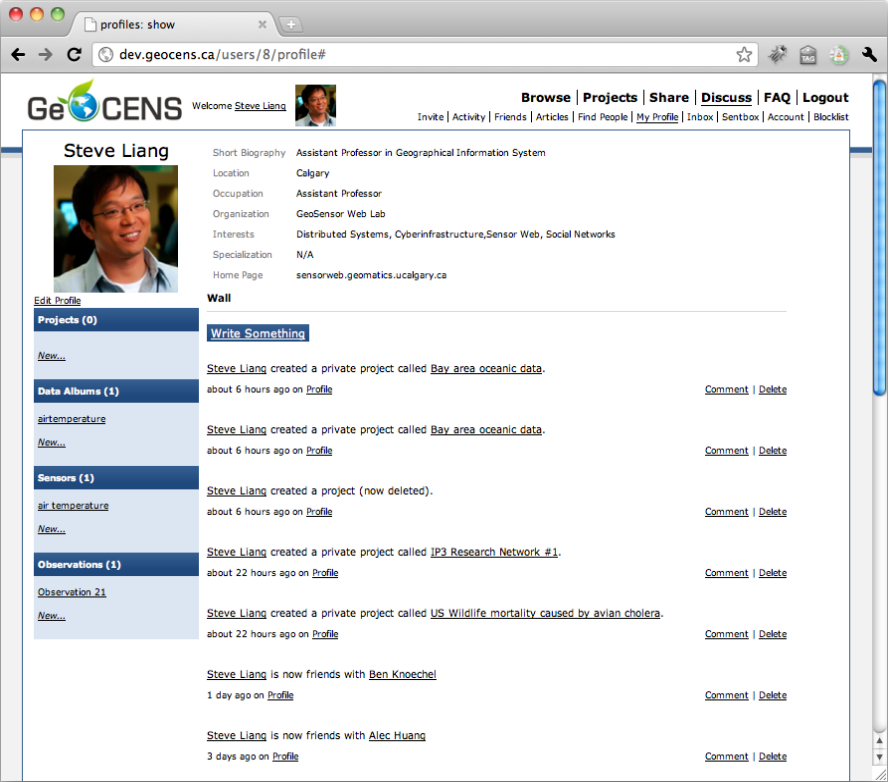
A screenshot of the GeoCENS online social network.

GeoCENS is an OSN-based sensor web platform for researchers. On GeoCENS, researchers can share sensors, scientific datasets, experiences, and activities with their friends (e.g., colleagues from other institutes) and social networks. GeoCENS users can create a profile where they declare their research interests and preferences, and establish friendships with other users. Figure 7 shows an example of the GeoCENS user profile. A “friendship” is formed on GeoCENS when one GeoCENS user sends a friendship invitation to another user. Upon confirmation by the latter, the friendship relationship is formed. Other features include the ability to: upload sensor datasets, join projects/groups with shared area of research interest. GeoCENS users have the ability to adjust different privacy levels, and review/annotate/rate sensors as well as datasets.

By creating a specialized OSN for sensor web users, our goal is to leverage the underlying social graphs, the structure of user interactions, and the users' profiles/preferences to create innovative uses and applications of the sensor web. One innovative OSN-based sensor web application is the sensor web recommendation engine.

### 4.5. Sensor Web Recommendation Engine

**Figure 8.**
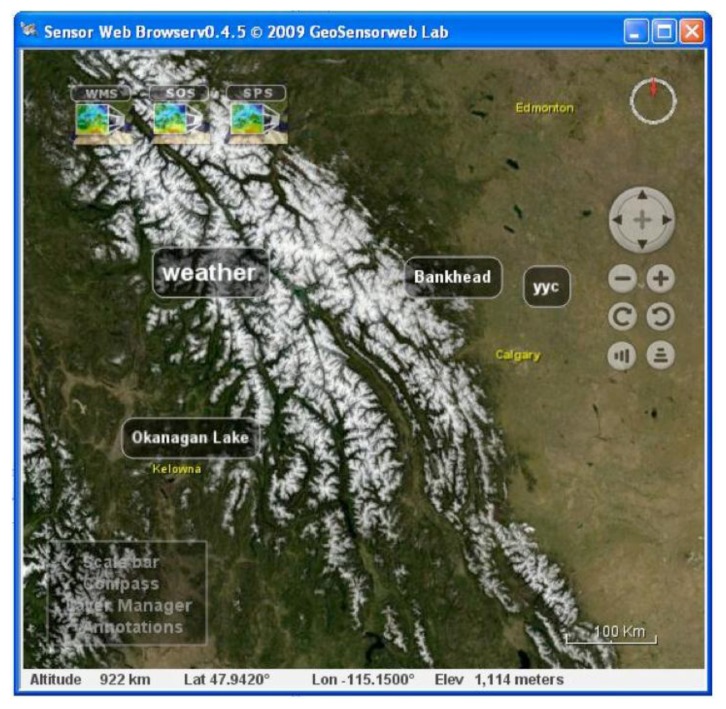
A screenshot of the GeoCENS recommendation engine tag map.

The GeoCENS social network infrastructure was used to develop a sensor web recommendation engine (*i.e.*, a collaborative tagging system) that recommends sensors and datasets according to a user's geographical area of interest. In fact, existing folksonomy-related research is mostly focused on non-geospatial applications [34]. One key contribution of the GeoCENS recommendation engine is that it extends the folksonomy research into geospatial applications. The recommendation engine leverages the geospatial information associated with three key components of collaborative tagging systems, namely, tags, resources, and users. The GeoCENS recommendation engine [35] provides the following three functions:

1. *Tag suggestion*: The recommendation engine enables users to assign tags to the resources. In order to make the task easier for the user and to avoid ambiguity, the recommendation engine suggests tags to the user. In a non-geospatial folksonomy system, when a user *u* is preparing to tag resource *r*, the recommended tags are ranked according to the weighted frequency of the tag *t*, for the resource *r*, using the following equation:
  (1) $$\text{Freg}(r,t,u)=\sum_{v\in \text{neig h}(u)}\text{Sim}(u,v)\left|\left\{(v,t,r)\in Y\right\}\right|$$
  where function *Sim*(*u*, *v*) is the similarity function between user *u* and his/her neighbor *v*, which is determined by the neighborhood function *neigh*(*u*) from the GeoCENS online social network. *Y* is the ternary relationship between user set *U*, tag set *T*, and resource set *R*, called the tag assignment. The term |{(*v*, *t*, *r*) ∈ *Y* }| evaluates to either 1 or 0 in a folksonomy, depending on whether the tag assignment exists or does not exist respectively.
  However, to consider the geospatial nature of sensors, the Tobler's first law of geography [36] was utilized, *i.e.*, “*Everything is related to everything else, but near things are more related than distant things*.” Therefore, Equation (1) is modified as follows:
  (2) $$\text{Freg}(r,t,u,p)=\sum_{v\in \text{neigh}(u)}\sum_{q\in \varphi (v,t,r)}\text{Sim}(u,v)\frac{\left|\left\{(v,t,r,q)\in Y\right\}\right|}{\text{Dist}(p,q)}$$
  where *p* is the geographical point at which tag *t* is inserted on this instance, *φ*(*v*,*t*,*r*) is the location of this tag assignment, and function *Dist*(*p*,*q*) is the distance function between points *p* and *q*. Thus, Equation (2) is the inverse distance weighted form of Equation (1).
2. *Tag browsing*: The tag browsing function enables users to navigate through the tags collected in the system. Tag browsing aids in the process of sensor and sensor data discovery. Considering the geospatial nature of tags, we have developed a new algorithm to calculate the font size and tag location when building the tag map. For the font size of a tag, we calculate the Freq score taking into consideration the distance from the center of the user's display (i.e., viewport). The equation for calculating font size is as follows:
  (3) $$\text{Freg}(t,u)=\sum_{r\in R}\sum_{v\in \text{neig h}(u)}\sum_{q\in \varphi (v,t,r)}\text{Sim}(u,v)\frac{\left|\left\{(v,t,r,q)\in Y\right\}\right|}{\text{Dist}(a,q)}$$
  where *a* is the center of the viewport. For the tag locations, first the distances from the center of the viewport to centroids of different tags are calculated. Then, the farthest tag is placed on the edge of the viewport. The locations of the other tags are calculated by normalizing with the distance between the viewport center and the farthest tag. Algorithm 1 presents the detail of calculating the tag positions. Figure 8 shows a screen capture of an example GeoCENS tag map.
**Algorithm 1.** Tag map calculation algorithm.**foreach**
*t* ∈ *T*
**do** Calculate centroid *c* of the set of all the points *u* ∈ *U*, *r* ∈ *R*, *φ*(u, t, r) Calculate distance *d_ac_* from eye-point *a* to *c***end** Calculate *d_max_*, the maximum of all *d_ac_***foreach**
*t* ∈ *T*
**do** Calculate *d_ab_*, the distance from point *a* to the point *b*, where line *ac* intercepts the viewport Calculate 
$d_{ag}=\frac{d_{ac}}{d_{\max}}d_{ab}$ Display tag *t* at *g*, where *g* is a point on line *ab*, and length of *ag* = *d_ag_***End**
3. *Tag searching*: The tag searching function enables users to retrieve resources based on tag queries, either by clicking on a tag, or by typing-in the tag. The key is to retrieve the most relevant resources for these queries. The proposed algorithm enhances the query processing by taking geospatial aspects of the queries and data into consideration. Similar to the Equation (3), the tag searching function takes the center of viewport and the locations of tags into consideration.

### 4.6. Semantic Layer Service

The sensor web is highly heterogeneous in terms of hardware, data types, observed phenomena, communication protocols, data encodings, semantics, syntactic, *etc.* Some of these heterogeneities can be addressed by applying open standard protocols, such as communication protocols and data encodings. However, even after applying open standards, there are interoperability issues from a lack of standardized naming, such as semantic heterogeneity and syntactic heterogeneity. As an example for semantic heterogeneity, consider the two strings “precipitation” and “rainfall”. Since rainfall is a type of precipitation, a user interested in precipitation data would likely be interested in rainfall. Although these concepts are intuitively related to any human, to any computer these are simply different sequences of characters.

The syntactic heterogeneity usually comes from the various labels used to represent the same concept, as different data providers may label their data differently. For example, Table 1 shows example of the various URIs used in SOS services to represent the same concept of wind speed. This syntactic heterogeneity causes difficulties to design a system to integrate all sensor data about wind speed.

Therefore, in order to address these heterogeneity issues, GeoCENS utilizes a semantic layer service, in which a bottom-up approach is used to integrate sensor data layers by their phenomena [37]. We first collect metadata from OGC SOS services (*i.e.*, capabilities documents). From the metadata, we extract atomic data layers known as Property Layers (PLs). While the names of PLs that convey information about the phenomenon they measure, these names are processed via normalization and tokenization text processing. Normalization is the process of canonicalizing strings such that superficial differences between strings are removed (e.g., “windspeeds” becomes “windspeed”). Tokenization is the process of converting text into distinct tokens (e.g., “windspeed” becomes “wind” and “speed”).

The similarity between two objects is a numerical measure of the degree to which the two objects are alike. Three similarity functions are used and compared for this work, a Length-Adjusted Levenshtein similarity function, the Jaccard similarity function, and a semantic similarity function. The Length-Adjusted Levenshtein similarity function is an edit-based function, while the latter two are set-based functions (for tokens).

The Length-Adjusted Levenshtein (LALD) similarity function is a modification of the Levenshtein distance [38]. It is used over the basic edit distance to reduce the impact of string length on the similarity between strings and normalize all similarity values between 0 and 1. The equation for calculating LALD similarity is as follows:

(4) $$d_{\text{LALD}}=\frac{d_{\text{Levens h tein}}}{\text{max}(|\text{string}1|,|\text{string}2|)}$$

The Jaccard coefficient is a measure of similarity between two data objects. Given two objects, the Jaccard coefficient is the number of shared binary attributes divided by the total number of binary attributes of both data objects. Therefore, this function requires the input of an array of tokens, where each token is a string. The equation for calculating LALD similarity is as follows:

(5) $$d_{\text{Jaccard}}=\frac{m_{11}}{m_{11}+m_{10}+m_{01}}$$

where *m_11_* is the number of words that exists in both strings, *m_10_* is the number of words that exist only in string 1, and *m_01_* is the number of words that exist only in string 2.

This semantic similarity function is based on the Jaccard similarity function using the set-based approach. We use WordNet as a lexical database to calculate word pair semantic similarity scores. The semantic similarity scores are used to (1) identify related word pairs and (2) assign weights to related word pairs. For more information, readers are referred to [37].

Using these similarity functions, we perform PL-PL mapping and PL clustering to find out the similarity between these sensor data instances. With the semantic layer service, the heterogeneous sensor data can be effectively integrated as well as provide a coherent view for users [32].

The Sensor Observable Registry (SOR) as one of the ongoing development in OGC SWE community also aims to address these heterogeneity issues [39]. SOR defines web service interfaces for users to retrieve (1) available defined URIs, (2) definition of a URI, and (3) semantically related URIs. As the GeoCENS semantic layer service mainly focuses on algorithms of grouping layers and constructing relations between layers with syntactic and semantic similarities of URIs, the outcome of the semantic layer service can be directly used by the SOR. Hence, we believe that the semantic layer service and SOR can complement with each other.

While the GeoCENS semantic layer service is able to provide a coherent view over heterogeneous sensor data, further research is needed to support fusion of different sensor data sets. For example, as the semantic layer service can answer simple queries like “what is the air temperature at place x and time t”, the service is not sufficient to answer complex queries such as queries that require combination of different data to infer events or higher-level facts. Semantics of sensor data can help to fuse heterogeneous sensor observations in a meaningful way [40].

## 5. Applications Powered by GeoCENS

**Figure 9.**
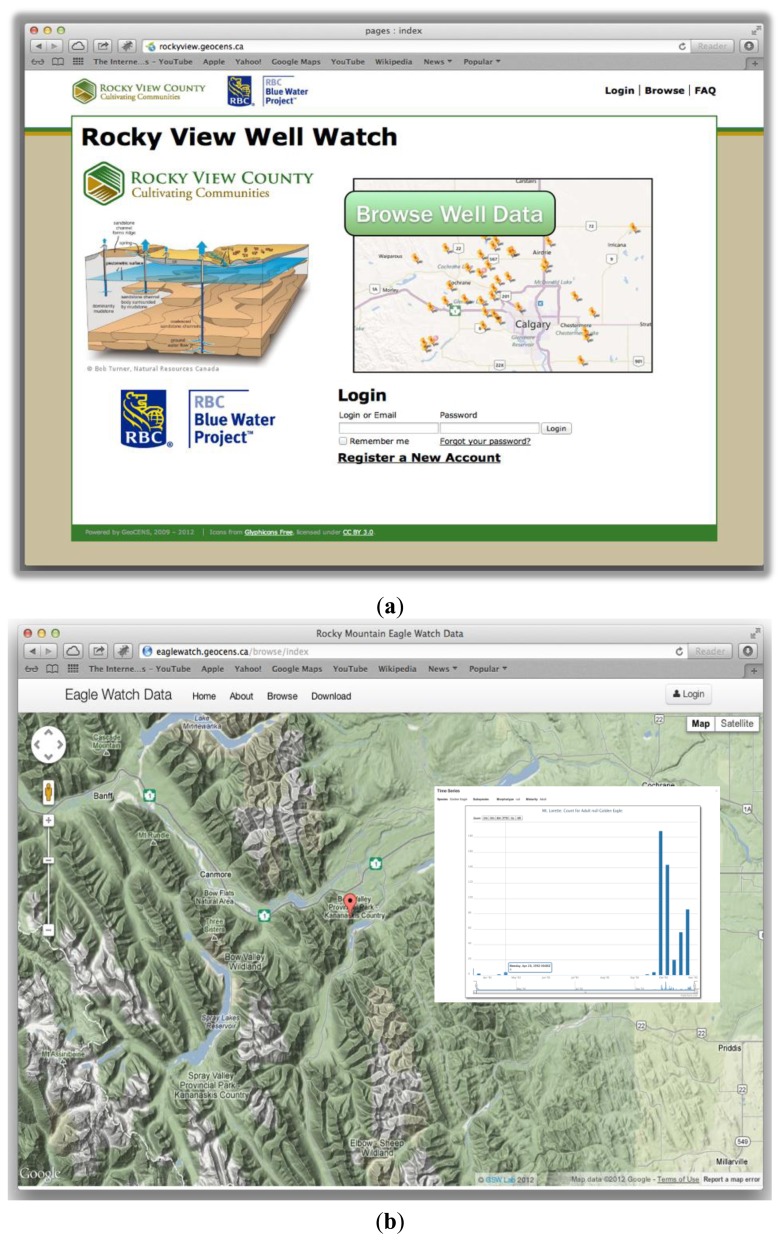

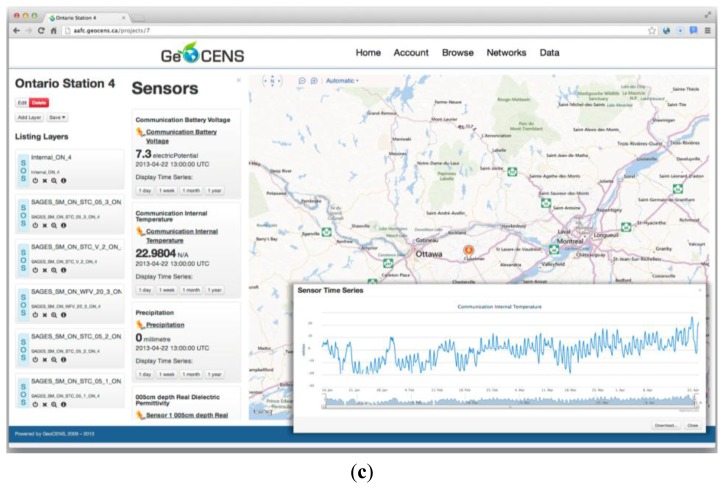
Screenshots of (**a**) Rocky View Well Watch, (**b**) Rocky Mountain Eagle Watch, and (**c**) Real-time In-Situ Monitoring for Agriculture (RISMA) projects.

As the GeoCENS project is designed for environmental researchers, we have also demonstrated that the GeoCENS architecture can be applied to many applications. For example, the *Rocky View Well Watch project* (http://rockyview.geocens.ca/) is a long-term groundwater monitoring project incorporating the Web 2.0 and VGI concept, where farmers can upload water level data of their water wells. Currently, the Rocky View Well Watch project has more than forty active water well owners, and the earliest data can be traced back to 2008. In addition, when the farmers upload their well readings, some hydrologists at the University of Calgary receive notifications and perform quality control (QC) processes. A screenshot of the Rocky View Well Watch project is shown in Figure 9a. As for the current status of the Rocky View Well Watch project, we are working with different municipalities to expand this well watch platform from a county-wide network to a nation-wide network.

The *Rocky Mountain Eagle Watch project* (http://eaglewatch.geocens.ca/) is another example that allows bird observers to submit the time and the location they observed a bird. The Rocky Mountain Eagle Watch is also a citizen sensing project for counting eagles for more than 20 years in the Rockies. In the past, the bird observers used papers and Excel data sheets to record and share their data, which is time-consuming and inefficient. By adapting the GeoCENS cyberinfrastructure architecture, the Rocky Mountain Eagle Watch platform has significantly simplified the data entry, analyzing, and sharing processes. Figure 9b shows a screenshot of the Rocky Mountain Eagle Watch project. However, as the identification of eagle species requires more training and expertise, the amount of data in this project does not grow as rapidly as the other two projects.

The third powered-by-GeoCENS project is the A*griculture and Agri-Food Canada*—*Real-Time In-Situ Soil Monitoring for Agriculture* (*RISMA*) (http://aafc.geocens.ca/). Soil moisture is a critical variable in agriculture environmental monitoring. Soil moisture is a determinant for rates of crop growth and productivity as well as rates of soil biogeochemical processes that impact soil fertility and determines boundary layer conditions that drive meteorological processes. In 2010 and 2011, Agriculture and Agri-Food Canada (AAFC), in collaboration with Environment Canada, established five in-situ monitoring networks. The networks were established near Kenaston (Saskatchewan), Carman (Manitoba), and Casselman (Ontario) as part of the Sustainable Agriculture Environmental Systems (SAGES) project titled Earth Observation Information on Crops and Soils for Agri-Environmental Monitoring in Canada. The near real time in-situ soil moisture/temperature and precipitation data from these five networks are used to calibrate and validate remote sensing and modeled soil moisture products. This project adapts the GeoCENS architecture to provide an interactive map of the network station locations and to view time series graphs of current and past data. Furthermore, users can sign up and log-in to download data series. Figure 9c shows a screenshot of the RISMA project. As for the status of the RISMA project, the number of sensors grows from 283 sensors in Ontario early 2013 to more than 1,408 sensors in three Canadian provinces in August 2013. We expect that this project will host more sensors in the near future and consequently becomes a Canadian monitoring network.

These *powered-by-GeoCENS* applications have already been opened to the public for one to three years and have demonstrated that the GeoCENS architecture can successfully provide an easy-to-use and interoperable cyberinfrastructure for sharing and visualizing sensor data from different domains. As GeoCENS follows the OGC open standards to host sensor data, the data collected by different applications are essentially *connected* in an interoperable manner. In addition, with the GeoCENS cyberinfrastructure, we can significantly reduce the cost of data collection and sharing (e.g., paper sheets, mail, travel, *etc.*), possibly leading to more efficient data processing and timely notification of abnormal events. Overall, as demonstrated by these three applications, we believe that the GeoCENS architecture can be applied to various domain applications. Consequently, GeoCENS is able to connect all sensors and corresponding data together to unleash more of the full potential of the world-wide sensor web.

## 6. Related Work

Several works have attempted to propose architecture for sensor web systems. Intel Research's IrisNet [41] proposes a decentralized architecture based on a hierarchical topology. IrisNet provides techniques to process queries over a distributed XML documents containing sensor data. However, IrisNet only supports very preliminary geospatial queries. Hierarchical place names are used to build its network topology. In order to perform geospatial query, users/applications need to know the exact place name a priori and explicitly specify the parts of a hierarchy that the query needs to traverse. Moreover, IrisNet does not have a sensor discovery module and only handles homogeneous sensors and data types.

Microsoft Research's SensorMap [19] uses a centralized web portal design and tackles the scalability and performance issues by building the COLR-Tree. The COLR-Tree is a data structure that indexes, aggregates, and caches sensor streams, in order to prevent transferring large volume of sensor streams across the network. However, SensorMap's centralized design makes the portal a single point of failure. In contrast, GeoCENS uses a Service-Oriented Architecture (SOA) and decentralized hybrid P2P architecture for sensor web service discovery. GeoCENS has no single point of failure, and the P2P sensor discovery service balances the load by directing queries and traffic to the distributed sensor web services.

The LiveWeb project [42] proposes a sensor web service portal to represent and monitor real-time data from sensors and other information providers. LiveWeb uses content-based publish/subscribe approach to provide real-time notifications of sensor data or events. However, all the approaches described above are based on proprietary service interfaces and sensor data encodings while GeoCENS follows OGC SWE specifications and is able to interoperate with other OGC-compliant sensor web clients and servers.

GeoCENS is not the only project applying OGC open standards on sensor web applications. 52° North (http://52north.org/) has been actively participating in OGC working groups and has developed and open-sourced many OGC SWE client and service implementations. The Center for Spatial Information Science and Systems (CSISS) in the George Mason University continues to participate in the OGC community over the last decade. In addition, [16,43] suggested SOA architectures and workflows that apply pure OGC solutions (including SOS, Sensor Planning Service, Sensor Event Service, Web Processing Service, Web Notification Service, *etc.*) to enable the discovery, exchange, and processing of sensor observations. However, these pure OGC approaches still have some issues. For example, [44,45] utilized Multi-Agent System approach to address identified service interaction and semantic interoperability issues. Similarly, in GeoCENS, we not only applied OGC standards but also proposed solutions (e.g., searching engine, LOST-Tree, and semantic layer service) to address issues that we identified in the real-world OWS services.

In GeoCENS, we try to propose an overall architecture by considering the necessary components in the sensor web. Although the presented GeoCENS architecture does not put much attention on the physical layer and sensor layer, we have been actively participating in the development of OGC standards for these layers (e.g., the PUCK protocol and the Sensor Web Interfaces for Internet of Things (SWE-IoT) standards) and trying to apply these standards into the GeoCENS architecture.

Furthermore, GeoCENS is innovative and unique in that it is a VGI-based and social network-based sensor web platform. GeoCENS harvests the sensor web users' interaction structures and activities in order to build innovative sensor web applications. With the embedded social network infrastructure, GeoCENS is able to build a geospatial folksonomy for the sensor web. This folksonomy recommends relevant sensor web resources to a user according to the collective intelligence of GeoCENS users.

## 7. Conclusions and Future Work

While the world-wide sensor web has become a very useful monitoring technique, we believe that the full potential of sensor web is not yet revealed. In this paper, we have analyzed the challenges and the components that must be developed to realize the vision of sensor web. We have identified the issue of long tail phenomenon on sensor web, and introduced our view of the sensor web architecture and its necessary components. Then, we have explained the GeoCENS architecture and proposed approaches to address different technical challenges. In addition, we have presented three powered-by-GeoCENS applications to demonstrate how the GeoCENS architecture can be applied to various domains and scenarios. By connecting all sensors and their data together, we believe the proposed architecture can consequently realize the world-wide sensor web vision.

We continue working on the GeoCENS architecture to make it more complete. For example, we have been actively participating in the development of OGC PUCK protocol and the Sensor Web Interfaces for Internet of Things (SWE-IoT) standards. These standards aim to improve the physical layer and sensor layer components. In addition, as the GeoCENS architecture tries to connect all sensors and their data together, one of the most important challenges is how to efficiently process the geospatial sensor data while the data are being produced at a high rate by a vast number of sensors. Therefore, we are also investigating the development of an event-driven process into the GeoCENS architecture to efficiently process the big sensor data and harvest the full potential of the sensor web. Furthermore, with the growing number of sensors being deployed by various organizations and individuals, issues such as privacy, security, as well as policy for sensor web will be one of our next focuses.

## Figures and Tables

**Table 1.** Various URIs of the concept of wind speed.

1	urn:x-ogc:def:property:OGC::WindSpeed
2	urn:ogc:def:property:universityofsaskatchewan:ip3:windspeed
3	urn:ogc:def:phenomenon:OGC:1.0.30:windspeed
4	urn:ogc:def:phenomenon:OGC:1.0.30:WindSpeeds
5	urn:ogc:def:phenomenon:OGC:windspeed
6	urn:ogc:def:property:geocens:geocensv01:windspeed
7	urn:ogc:def:property:noaa:ndbc:Wind Speed
8	urn:ogc:def:property:OGC::WindSpeed
9	urn:ogc:def:property:ucberkeley:odm:Wind Speed Avg MS
10	urn:ogc:def:property:ucberkeley:odm:Wind Speed Max MS
11	http://marinemetadata.org/cf#wind speed
12	http://mmisw.org/ont/cf/parameter/winds
